# Heroin Adulterated with the Novel Synthetic Cannabinoid, 5F-MDMB-PINACA: A Case Series

**DOI:** 10.5811/cpcem.2020.2.45060

**Published:** 2020-04-23

**Authors:** Muhammed Ershad, Maricel Dela Cruz, Ahmed Mostafa, Muhammad M. Khalid, Ryan Arnold, Richard Hamilton

**Affiliations:** * Drexel University College of Medicine, Department of Emergency Medicine, Division of Medical Toxicology, Philadelphia, Pennsylvania; † Drexel University College of Medicine, Department of Emergency Medicine, Philadelphia, Pennsylvania

**Keywords:** synthetic cannabinoids, heroin, physostigmine

## Abstract

**Introduction:**

Heroin can be adulterated with various substances that may or may not have pharmacological effects. Here we report a case series of 8 patients who presented to the emergency department after overdose with intravenous heroin preparation adulterated with the synthetic cannabinoid methyl 2-(1-(5-fluoropentyl)-1H-indazole-3-carboxamido)-3,3-dimethylbutanoate (5F-MDMB-PINACA).

**Case Series:**

Except for one patient, all of them presented with a typical initial opioid toxidrome consisting of central nervous system and respiratory depression along with pinpoint pupils. Naloxone was given to them, triggering severe agitation and combative behavior along with overlapping features of anticholinergic and sympathomimetic toxidrome. All patients required multiple doses of benzodiazepines. Three were successfully treated with physostigmine.

**Discussion:**

5F-MDMB-PINACA is a synthetic cannabinoid that was added to heroin in samples obtained from patients reported in this case series. Patients demonstrated significant agitation after receiving naloxone for opioid toxidrome, presumably because of the removal of the depressant effect of opioids, which unmasked the excitatory effects of the synthetic cannabinoids. Three patients required physostigmine along with the benzodiazepines for control of their agitation, urine retention and abnormal vitals, suggesting the possibility of an anticholinergic toxidrome to have developed in these patients.

**Conclusion:**

Heroin contaminated with 5F-MDMB-PINACA exhibits variable severities of anticholinergic effects, some on presentation and others only after opiate antagonism.

## INTRODUCTION

Heroin is often adulterated with a variety of substances including baking soda, caffeine, crushed analgesics, and scopolamine.^1^ We present a consecutive patient case series with similar presentations after overdose with intravenous (IV) heroin adulterated with the synthetic cannabinoid (SC) methyl 2-(1-(5-fluoropentyl)-1H-indazole-3-carboxamido)-3,3-dimethylbutanoate (5F-MDMB-PINACA). Within three months, eight patients presented to the emergency department (ED) after use of IV heroin with symptoms consistent with anticholinergic toxicity and variable requirement for naloxone administration by emergency medical services (EMS).

## CASE SERIES

### Case 1

**Image 1:**
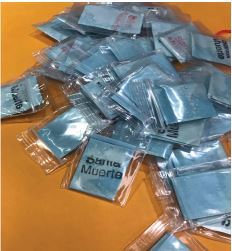
Packets of “Santa Muerte,” a street heroin adulterated with synthetic cannabinoid, that were retrieved from a patient found unresponsive at home.

A 28-year-old man with a past medical history of bipolar disorder and polysubstance abuse including IV heroin, presented to the hospital by EMS after being found at home unresponsive. Family members found packets of drugs near the patient labelled “Santa Muerte (Image 1). On arrival, vital signs included a heart rate (HR) of 122 beats per minute, blood pressure (BP) 134/78 millimeters of mercury (mm Hg), respiratory rate (RR) of 38 breaths per minute, oral temperature 98.2 degrees Fahrenheit (F), and oxygen saturation (SpO2) 78% on non-rebreather mask. Physical exam included tachycardia, flushing, dry mucous membranes and mydriasis. The patient was initially given two milligrams (mg) of intranasal (IN) naloxone in the field by EMS secondary to central nervous system (CNS) and respiratory depression, with no response. He was given a second dose of two mg IN naloxone and became agitated and combative. The patient was intubated upon ED arrival for hypoxic respiratory failure. Chest radiograph showed signs of aspiration pneumonitis, which developed into acute respiratory distress syndrome (ARDS) requiring venovenous extracorporeal membrane oxygenation (VV-ECMO). Head computed tomography was negative for acute intracranial abnormality. Complete blood count (CBC) and basic metabolic panel (BMP) were unremarkable. Urine drug screen immunoassay was positive for cocaine, opiates, fentanyl, tetrahydrocannabinol (THC) and benzodiazepines. Comprehensive drug screen of the serum by liquid chromatography tandem mass spectrometry (LC-MS-MS) was positive for cocaine, heroin, 6-monoacetylmorphine (6-MAM), fentanyl, THC, and alprazolam. The patient remained intubated on VV-ECMO for 12 days, after which he was extubated, removed off of VV-ECMO, and discharged on day 17. Laboratory analysis of the patient’s confiscated drug by gas chromatography-mass spectrometry (GC-MS) and liquid chromatography quadrupole time-of-flight mass spectrometry (LCQ-TOF-MS) was positive for the novel SC 5F-MDMB-PINACA, heroin, and fentanyl.

**CPC-EM Capsule**

What do we already know about this clinical entity?

Synthetic cannabinoids (SC) may be added as adulterants to opiates sold on the street, which can contribute to unpredictable clinical consequences.

What makes this presentation of disease reportable?

We report a case series of eight patients who had predominantly anticholinergic features after using heroin containing the SC 5F-MDMB-PINACA.

What is the major learning point?

Patients using heroin containing SCs may exhibit severe agitation and hyperactive behavior following naloxone administration.

How might this improve emergency medicine practice?

Consider using physostigmine along with benzodiazepines in treating patients with severe agitation following naloxone-induced reversal of an opioid toxidrome.

### Case 2

A 25-year-old man with a past medical history of IV heroin use, presented to the ED by EMS after IV heroin use. The patient initially had CNS and respiratory depression in the field and was first given two mg of IN naloxone with no response, followed by a second dose of two mg IN naloxone, which made him anxious and tachycardic. Vital signs on arrival to the ED included a HR of 102 beats per minute, BP of 146/89 mmHg, RR 24 breaths per minute, SpO2 98% on room air, and oral temperature 97.5º F. Physical exam was positive for flushing, tachycardia, and agitation. The patient was given 4 mg of lorazepam IV in the ED. He admitted to the use of an adulterated heroin “Santa Muerte.” CBC and BMP were unremarkable. Urine drug screen immunoassay was positive for opiates, amphetamine, barbiturates and cocaine. Symptoms improved after benzodiazepine treatment, IV fluids, and supportive care. He was admitted for 24 hours and discharged the following day with no further complications. Laboratory analysis of the patient’s confiscated drug by GC-MS and LCQ-TOF was positive for the novel SC 5F-MDMB-PINACA, heroin, and fentanyl.

### Case 3

A 31-year-old man with a past medical history of IV heroin use, presented to the ED by EMS for CNS and respiratory depression after IV heroin use. The patient’s girlfriend provided the history that the patient was using a new type of heroin called “Santa Muerte.” The patient was given a total of four mg IN naloxone in the field, after which he became agitated, combative, and tachycardic. His vital signs on arrival included a HR of 163 beats per minute, BP of 131/81 mmHg, RR of 29 breaths per minute, SpO2 99% on room air, and oral temperature 98.8 degrees F. While in the ED, he continued to be agitated and combative. On examination, he was tachycardic and flushed with dilated pupils and a palpable full bladder in the suprapubic region. The patient was given a total of 10 mg of lorazepam with minimal improvement of his agitation, and he was later intubated for airway protection. Complete blood count (CBC) and basic metabolic panel (BMP) were unremarkable, and urine drug screening immunoassay was positive for opiates. Serum comprehensive drug screen by LC-MS-MS was positive for heroin, 6-MAM, fentanyl, and negative for any SCs. The patient later developed ARDS, requiring increased ventilator setting and was transferred to a tertiary center for VV-ECMO. Specialty laboratory testing of the patient’s confiscated drug by GC-MS and LCQ-TOF was positive for the novel SC 5F-MDMB-PINACA, heroin, and fentanyl.

### Case 4

A 25-year-old man presented to the hospital by EMS after IV heroin use. The patient was found with a drug packet labeled “Santa Muerte” in his pocket and had CNS and respiratory depression. He was given a total of four mg of IN naloxone, after which he became flushed, tachycardic, and agitated with dilated pupils. On arrival to the ED, his HR was 158 beats per minute, BP was 215/158 mmHg, RR was 26 breaths per minute, SpO2 was 99% on room air, and oral temperature was 102.1º F. On exam, he had urinary retention and anhidrosis. He was given four mg of lorazepam and two mg of IV physostigmine, which treated his agitation. There was also marked improvement in anhidrosis and urine retention. He was admitted for 24 hours and discharged the following day with no further complications. Urine drug screen was positive for cocaine, opiates, and THC. Serum comprehensive toxicology analysis by LC-MS-MS was positive for 5F-MDMB-PICA(5F-ADB), heroin, 6-MAM, and fentanyl. Laboratory analysis of the patient’s confiscated drug by GC-MS and LCQ-TOF was positive for the novel SC 5F-MDMB-PINACA, heroin, and fentanyl.

### Case 5

**Image 2:**
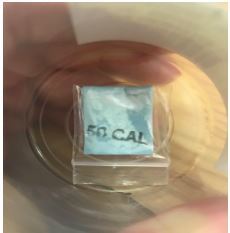
A “50 CAL” drug packet retrieved from a patient, which contained heroin, synthetic cannabinoid, and fentanyl.

A 45-year-old man was found down in the field agitated and tachycardic. On arrival to the ED, his HR was 124 beats per minute, BP 140/82 mm Hg, RR 22 breaths per minute, oxygen saturation 99%, and oral temperature 99.3º F. On exam, he had pinpoint pupils with flushing of skin. He received midazolam five mg and olanzapine 20 mg intramuscular followed by diazepam 10 mg IV after which he calmed down. He was eventually started on dexmedetomidine infusion when his agitation returned. He was admitted for 24 hours and discharged the following day with no complications. Urine drug screen was positive for opiates and fentanyl. He was found with a drug packet named “50 CAL” (Image 2), which was sent for GC-MS and LCQ-TOF and was found to be positive for 5F-MDMB-PINACA, heroin, and fentanyl.

### Case 6

A 36-year-old man was found lying in the street unresponsive. He received eight mg of naloxone IN after which he became agitated. On arrival to the ED, his HR was 130 beats per minute, BP 160/100 mm Hg, RR 24 breaths per minute, oxygen saturation 95% on 100 % oxygen, and oral temperature 98.6º F. Initial physical examination revealed restlessness, confusion, and picking behavior. Patient also had bilaterally dilated pupils with urine retention on point-of-care ultrasound. Considering an anticholinergic toxidrome, the emergency provider administered physostigmine two mg IV with improvement in agitation, picking behavior, urine retention, and relative constriction in pupillary diameter. He had received multiple doses of benzodiazepines prior to physostigmine. He was eventually intubated due to risk of aspiration from vomiting in the setting of altered mental status. His mental status and vitals improved the next day, following which he was extubated. CBC and BMP were unremarkable and urine drug screen was positive for opiates and fentanyl. The patient was found with a blue packet labeled “50 CAL,” which was found to be positive for fentanyl, heroin, and 5F-MDMB-PINACA on GC-MS and LCQ-TOF.

### Case 7

A 23-year-old woman was brought to the ED with severe agitation and combative behavior. Her initial vitals were HR 156 beats per minute, BP 147/64 mm Hg, RR 20 breaths per minute, and oral temperature 101.5º F. Examination revealed bilaterally dilated pupils, flushed and dry skin, and urine retention on point-of-care ultrasound. The patient received Lorazepam four mg IV and physostigmine two mg IV after which her agitation subsided, urine retention improved, pupillary diameter decreased, and skin appeared less flushed and less dry. CBC and BMP were unremarkable while her urine drug screen was positive for opiates and fentanyl. She was admitted to the floor and discharged the next day. The patient reported consuming a substance from packets labeled “50 CAL.” The drug packets were not available for analysis. We were also unable to send her serum or urine for further comprehensive toxicology analysis.

### Case 8

A 27-year-old man was brought to the ED after IV heroin use. He was found to be in respiratory and CNS depression with pinpoint pupils in the field by the EMS. Naloxone four mg IN was given after which he became agitated. His vitals were HR 130 beats per minute, BP 130/94 mm Hg, RR 22 breaths per minute, and temperature of 99º F. Initial examination revealed dilated pupils, dry oral mucous membrane, and flushed skin. He received lorazepam 4 mg IV and physostigmine two mg IV after which he calmed down, pupils returned back to normal size, and heart rate came down to normal; he was admitted to the floor. Urine drug screen was positive for opiates and fentanyl. He reported having ingested drugs from packets labeled “Nick” and “50 CAL,” but they were unavailable for analysis.

## DISCUSSION

Heroin has been historically adulterated with a variety of substances including baking soda, caffeine, acetaminophen, diphenhydramine, scopolamine, fentanyl, and clenbuterol.^1^ These adulterants are usually added to increase profits by incorporating any substance that looks like the original substance and/or would have the same effect. In the months of April and August 2018, consumption of heroin that had been laced with the newer SC 5F-MDMB-PINACA gave rise to a series of patients presenting to our ED with unique clinical manifestations.

SCs, by themselves, have been widely used as drugs of abuse since the early 2000s. They have been found to have more adverse clinical presentations than the active compound marijuana itself, owing to its full agonistic action on the cannabinoid receptor type 1 (CB1) and cannabinoid receptor type 2 (CB2) receptors, as compared to marijuana, which is only a partial agonist.^2^ Clinical effects of SC overlap with anticholinergic and sympathomimetic toxidromes.^3^,^4^ There have also been reported fatalities with SCs including 5F-ADB, 5F-PB-22, and AB-CHMINACA.^5^

The patients reported in our case series took opioids containing 5F-MDMB-PINACA (Figure), which is a new generation SC. Except for patient 5, all of them presented with a typical initial opioid toxidrome consisting of CNS and respiratory depression along with pinpoint pupils. Naloxone, an opioid antagonist, was given to them, which triggered severe agitation and combative behavior along with overlapping features of anticholinergic and sympathomimetic toxidrome. This was presumably because of the removal of the depressant effect of opioids by the administration of naloxone that unmasked the effects of SC.

All the patients required multiple doses of benzodiazepines. Three of the eight patients were successfully treated with physostigmine, which helped control the abnormal psychomotor activity and anticholinergic manifestations.

The initial urine drug screen test used for all eight patients was an immunoassay-based screening test. Apart from morphine (opiates), it tests for fentanyl, buprenorphine, methadone, tramadol, cocaine, oxycodone, phencyclidine, amphetamines, barbiturates, benzodiazepines, and cannabinoids. The comprehensive serum drug screen performed in cases 1, 3 and 4 was through LC-MS-MS, which is an exceedingly sensitive and specific analytical technique that can precisely estimate the identities and concentrations of molecules within a sample.

The seized drug packets were analyzed at the Center for Forensic Science Research and Education, using GC-MS and LCQ-TOF. The samples were prepared using acid/base extraction prior to the analysis.^6^ The drug packets that were analyzed at this facility did not turn positive for any of the anticholinergic agents, thereby leading us to conclude that 5F-MDMB-PINACA potentially has anticholinergic manifestations that seem to be responding to physostigmine in our clinical experience.

## CONCLUSION

Heroin contaminated with 5F-MDMB-PINACA exhibits variable severities of anticholinergic effects, some on presentation and others only after opiate antagonism. Synthetic cannabinoids affect cannabinoid CB1 and CB2 receptors, potentially causing adrenergic stimulation, sedation, hallucinations, catecholamine release, and severe tachycardia.^2^–^4^ It is also possible that synthetic cannabinoids and/or their metabolites interact directly with acetylcholine receptors to cause anticholinergic effects. In our case series, we found that physostigmine was effective in reversing the anticholinergic effects and agitation in three out of the eight patients.

## Figures and Tables

**Figure:**
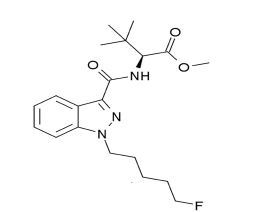
Chemical structure of the synthetic cannabinoid methyl 2-(1-(5-fluoropentyl)-1H-indazole-3-carboxamido)-3,3-dimethylbutanoate.
